# Pulp Revascularization in an Autotransplanted Mature Tooth: Visualization with Magnetic Resonance Imaging and Histopathologic Correlation

**DOI:** 10.3390/jcm12186008

**Published:** 2023-09-16

**Authors:** Petra Rugani, Iva Brcic, Marton Magyar, Uwe Yacine Schwarze, Norbert Jakse, Kurt Ebeleseder

**Affiliations:** 1 Department of Dental Medicine and Oral Health, Division of Oral Surgery and Orthodontics, Medical University of Graz, Billrothgasse 4, 8010 Graz, Austria; 2 Diagnostic and Research Institute of Pathology, Comprehensive Cancer Centre Graz, Medical University of Graz, 8010 Graz, Austria; iva.brcic@medunigraz.at; 3 Department of Radiology, Division of Neuroradiology, Vascular and Interventional Radiology, Medical University of Graz, 8010 Graz, Austria; marton.magyar@medunigraz.at; 4 Department of Dentistry and Oral Health, Division of Oral Surgery and Orthodontics, Medical University of Graz, 8010 Graz, Austria; uwe.schwarze@medunigraz.at; 5 Department of Orthopedics and Traumatology, Musculo-Skeletal Research Unit for Biomaterials, Medical University of Graz, 8036 Graz, Austria; 6 Department of Dental Medicine and Oral Health, Division of Prosthodontics, Restorative Dentistry and Periodontology, Medical University of Graz, 8010 Graz, Austria; kurt.ebeleseder@medunigraz.at

**Keywords:** autotransplantation, magnetic resonance imaging, cone-beam computed tomography, histopathology

## Abstract

Autotransplantation of a mature tooth usually leads to pulpal necrosis. Root canal treatment is recommended to prevent related inflammatory complications a few weeks after surgery. Extraoral root-end resection may facilitate reperfusion and obviate root canal treatment, but cannot be pictured with conventional dental radiography at this point in time. In the case of a lower mature transplanted molar, contrast-enhanced magnetic resonance imaging proved to be a feasible method for visualizing pulp revascularization just 4 weeks after autotransplantation. Consequently, root canal treatment was obviated. Nevertheless, the tooth had to be extracted 18 months postoperatively due to external cervical root resorption, probably caused by the extraction trauma. This allowed the histological processing and examination of the newly generated intracanal tissue. Uninflamed fibrovascular connective tissue was found, while odontoblasts or cementoblast-like cells were absent. These findings indicated that it was most likely stem cells from the bone marrow and the periodontal ligament that drove the regeneration.

## 1. Introduction

Tooth autotransplantation (AT) is defined as repositioning of a tooth or tooth germ in the same patient’s mouth. It has been an accepted method since the 1980s. Success is defined by two main aspects: Periodontal and endodontic healing of the transplant at the recipient site.

A prerequisite for periodontal healing is a gentle surgical technique. Especially, damage of the root cementum has to be avoided. Thorough preoperative planning and the application of surgical templates have proven to be beneficial [1]. Endodontic healing after AT is defined as an ingrowth of new tissue through the apical foramen into the pulp canal and pulp chamber of the transplant (pulp revascularization, PRV). PRV in immature transplanted teeth with a developing root is common and is probably facilitated by the large diameter of the apical foramen and the high number of apical stem cells [2,3]. A small diameter of 0.2–0.4 mm however, as found in the apical constrictions of mature premolars, reduces the chances of PRV to almost zero [4]. As a failed PRV is mostly combined with endodontic infection, rapid external infection-related root resorption can follow. Consequently, in mature teeth root canal treatment is recommended within 7–14 days after AT [5,6].

Assessment and evidence of successful PRV is therefore a critical issue for further treatment of autotransplanted teeth. Two aspects have to be considered: First, successful PRV should not be disturbed by an unnecessary endodontic intervention and second, root resorption has to be avoided in cases of failed PRV. Traditionally, PRV is assessed indirectly. First by ruling out necrosis (i.e., lack of signs indicating infection related root resorption) and later by pulp canal obliteration (PCO) on intraoral radiographs and/or clinically by the regained sensitivity of the tooth.

A more recent approach aims to show PRV directly via magnetic resonance imaging (MRI) with or without the use of a contrast agent [7,8,9,10]. In mature teeth, with a high risk of pulp necrosis, this verification may obviate the need for prophylactic root canal treatment several weeks after surgery.

Several case reports [11,12] and recent studies [13,14] indicated that extra-oral root end resection (EORER) might promote PRV in autotransplanted teeth and thus prevent endodontic complications. We hereby report for the first time on histological findings that might help to understand the biological process underlying the PRV of autotransplanted apically resected mature teeth (ATMT-EORER). Contrast-enhanced MRI was applied for non-invasive success control.

## 2. Report

The PRICE reporting guidelines were followed in the preparation of this report [15].

**Figure 1:**
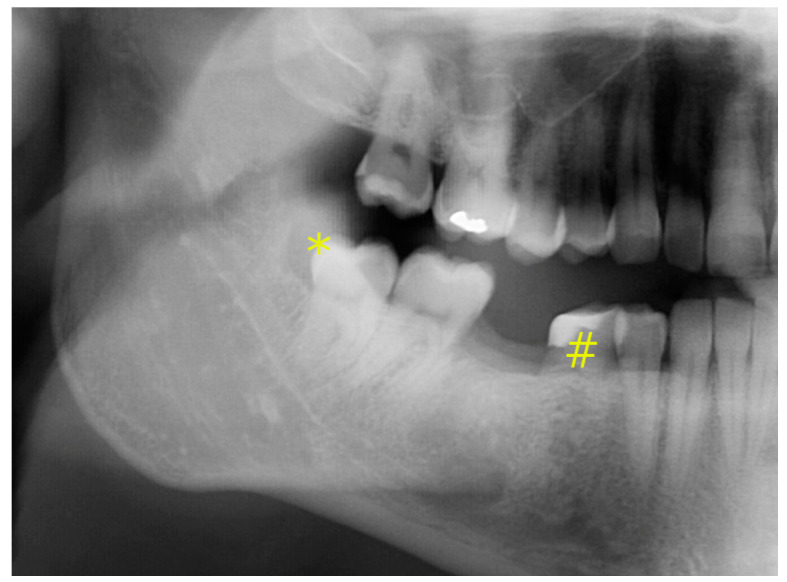
Initial panoramic radiograph; * Signs for pericoronitis tooth 48 as indication for tooth removal; # Deciduous tooth 85 with heavy restoration and severely resorbed roots and a poor long-term prognosis.

A 28-year-old male white patient was referred by his orthodontist with pericoronitis at the partially impacted lower right third molar. Removal of tooth 48 (FDI (World Dental Federation)) and orthodontic therapy to resolve crowding was intended. In his past medical history, he had lost the left lower first molar three years prior to admission due to apical periodontitis. Due to aplasia of tooth 45, the deciduous tooth 85 was still present but its roots were severely resorbed (Figure 1). In the course of the treatment planning, the decision was made to transplant the partially impacted tooth 48 in the position of the missing second premolar as an alternative to implant placement.

The patient was in good general health but was a smoker. He gave informed consent to autotransplantation, knowing that prognosis was reduced due to the mature multi-rooted donor tooth.

**Figure 2:**
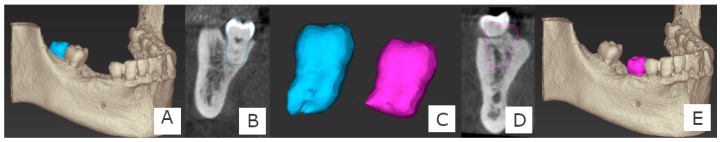
(**A**,**B**) Segmentation of the tooth transplant (blue) (**C**) segmented donor (blue) and planned transplant after EORER (pink) (**D**,**E**) Planned transplant at the recipient site (pink).

In the preoperative analysis, a CBCT (Cone beam computed tomography) scan was obtained using Planmeca ProMax 3D Max (Planmeca OY, Helsinki, Finland), with a voxel size of 0.2 mm and standard settings to confirm the integrity of the donor tooth and adequate dimensions of the recipient site. CBCT data were then segmented using the coDiagnostiX^TM^ software (Version 9.0, Dental Wings GmbH, Chemnitz, Germany) to create a virtual 3D replica of the tooth transplant. This model was 3D printed to serve as a reference for recipient-site preparation (Figure 2).

**Figure 3:**
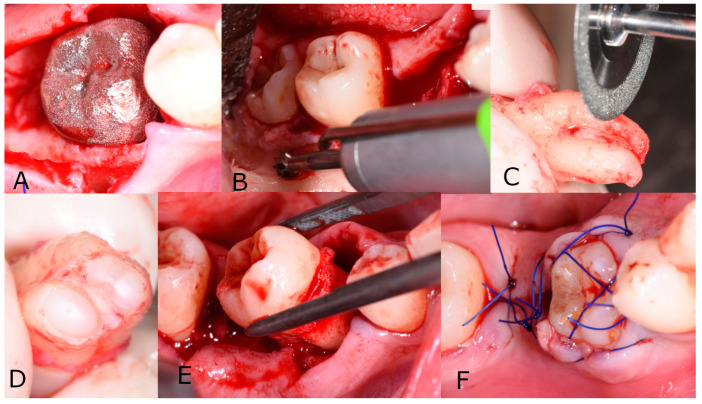
Autotransplantation (**A**) Tooth replica fitted in the prepared new socket (**B**) Osteotomy to remove the graft tooth 48 (**C**) Root-end resection of the transplant (**D**) Enlarged apical opening (**E**) Re-insertion of the graft into the new socket (**F**) Wound closure.

**Figure 4:**
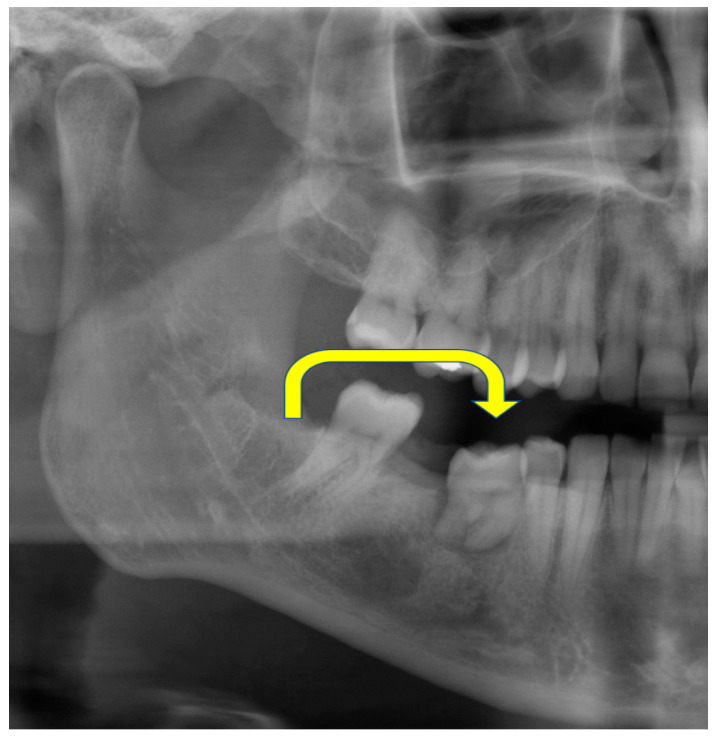
Postoperative panoramic radiograph. After removal of the deciduous tooth 85 the wisdom tooth and surgical preparation of the recipient socket tooth 48 had been transplanted (Yellow arrow). Note the shorter roots of the transplant after EORER.

The surgery was performed under local anesthesia (Ultracain Dental Forte 1:100,000; Sanofi-Aventis GmbH, Vienna, Austria). Tooth 85 was removed and a local mucoperiosteal flap was raised at the recipient site. The recipient socket was prepared using an implant drill kit (Frialit II, Dentsply Sirona Austria GmbH, Vienna, Austria). Sufficient enlargement was verified using the printed graft replica. (Figure 3A) Thereafter, pericoronal osteotomy was performed (Figure 3C) and tooth 48 was extracted as gently as possible. Care was taken not to damage the root cement, but because of the impaction elevators had to be used as well. The root tip was resected (EORER, Figure 3C) with 40,000 rpm under water cooling with a diamond disc. As a result, the apical opening increased to >1 mm. (Figure 3D) There was no additional extracorporeal treatment of either pulp or cementum. Subsequently, the graft was gently inserted into its new socket. (Figure 3E and Figure 4) After insertion, the flap was closed and the transplant was fixed in the socket using sutures (Figure 3F).

A flexible wire-composite splint was bonded to the vestibular enamel of the transplant and the neighboring teeth. The metallic component of the splint was a twistflex wire (GAC Wildcat Wire 0.0175-inch, Ortho-Care, Shipley, UK).

The patient received perioperative antibiotic prophylaxis for 4 days (cephalexin administered thrice daily for 4 days; Ospexin^®^ 1000 mg, Sandoz GmbH, Kundl, Austria) and antiphlogistic therapy was prescribed for 2–5 days (dexibuprofen administered thrice daily for 2–3 days; Seractil^®^ forte 400 mg, Gebro Pharma GmbH, Fieberbrunn, Austria). Sutures were removed after 1 week, the wire splint after 4 weeks.

The patient was recalled every three months during the first year. The endodontic status of the transplant was assessed during each follow-up. The following clinical endodontic data were obtained:

- Presence/absence of local swelling or sinus tract
- Sensitivity to percussion
- Pulpal sensitivity to an electric pulp tester (Digitest^®^, Parkell, NY, USA).

**Figure 5:**
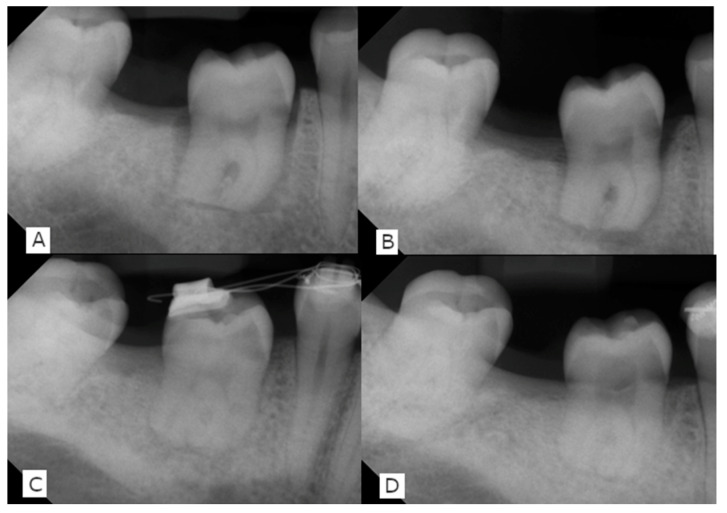
Radiographic follow-up with intraoral radiographs. (**A**) postop (**B**) 3 months postop beginning formation of the periodontal ligament space, no signs for external root resorption (**C**) 12 months postop, visible obliteration of the pulp chamber (**D**) 18 months postop radiolucent lesion in the pulp chamber.

Intraoral radiographs were obtained using the rectangular technique with appropriate film holders. The presence of pulp obliteration (a sign of revascularization), external infection-related root resorption, or apical radiolucency (signs of infected pulp necrosis) were investigated (Figure 5).

**Figure 6:**
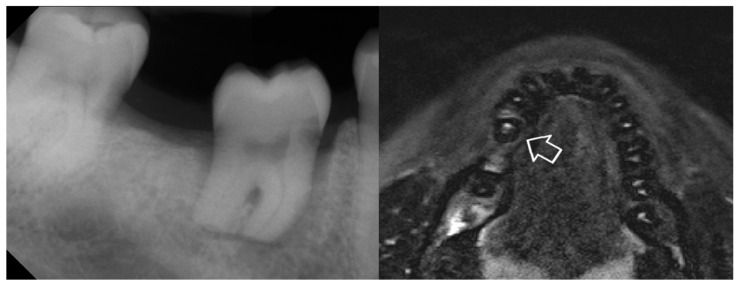
Radiographic control 4 weeks postoperatively. (**Left**), intraoral radiograph with still-missing signs of PDL regeneration or reperfusion. (**Right**), contrast-enhanced MRI with uptake of the contrast medium in the pulp chamber (arrow) already confirming reperfusion.

Contrast-enhanced magnetic resonance imaging (MRI) was performed immediately after splint removal to assess revascularization using a contrast medium (Gadobutrol, Gadovist^®^, Bayer, Leverkusen, Germany; Figure 6 right). 3D T1 and T2 sequences were obtained with and without contrast agent to detect the accumulation of the contrast agent (Siemens Magnetom Prismafit, T1 Starvibe 0.7 mm 3D with contrast agent). The good local resolution allowed a significant magnification of the transplanted teeth and thus, an assessment of the small volume pulp in all three dimensions. A head and neck radiologist with more than 10 years of experience was responsible for the interpretation of the images.

Electromechanical tapping (Periotest^®^, Medizintechnik Gulden, Modautal, Germany) was performed to assess tooth mobility and values were compared to neighboring corresponding teeth, respectively. Periotest values were similar and were slightly positive after 12 months (+3).

The postoperative course was uneventful. The MRI showed enhancement of the contrast agent in the pulp chamber four weeks postoperatively (Figure 2B) and sensitivity was restored three months after surgery. Orthodontic therapy was initiated after the 9-month-recall with the transplanted tooth engaged in tooth movement.

**Figure 7:**
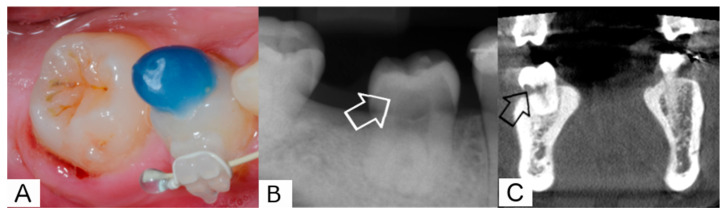
18-month follow-up. (**A**). Clinical picture with signs of inflammation (bleeding and suppuration from the gingival margin) (**B**). Intraoral radiograph with suspect radiolucency in the crown of the transplant (white arrow) (**C**). Native CBCT (resolution 0.2 mm) The arrow shows the buccal resorption in the subgingival part of the crown of the transplanted tooth, but otherwise a regular periodontal ligament space and no further signs of external root resorption.

After 18 months, the patient presented with a buccal swelling with purulent drainage (Figure 7A) over the cervical aspect of the transplant. The CBCT scan revealed external resorption in the subgingival part of the crown, especially the dental neck (Figure 7C). As a consequence of its reduced prognosis, the tooth was extracted with the patient’s consent.

The extracted tooth was fixed in 4% phosphate-buffered formaldehyde pH 7 immediately after extraction. After this, the tooth was rinsed in tap water then dehydrated in ascending grades of ethanol (40%, 70%, 80% 96% 3 times absolute for analysis). Infiltration with a light curing resin (Technovit 7200, Kulzer, Hanaus, Germany) was performed in ascending grades mixed with ethanol absolute for analysis (30:70, 50:50, 70:30, 3 times pure Technovit 7200). Finally, the tooth was embedded in the same resin and processed into undecalcified thin ground sections with a thickness of about 100 µm, according to the Karl Donath method [16], using machines from EXAKT (EXAKT Apparatebau, Norderstedt, Germany) and Walter Messner (Walter Messner GmbH, Oststeinbek, Germany). A section was stained with basic fuchsin methylene blue and Azur II [17].

**Figure 8:**
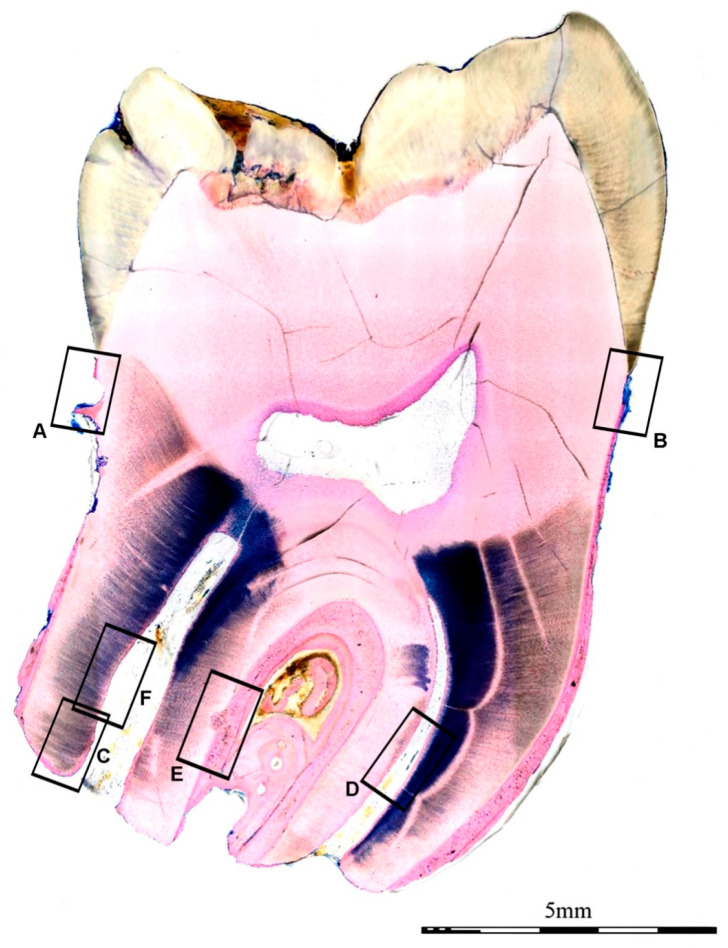
Low power image of an explanted/extracted tooth 18 months after transplantation. The enamel is ulcerated. Resorptive lacunae and cementum damage are visible at the root surface. The canal lumen is composed of vascularized fibro-connective tissue. Bony islands are also present. Details marked with rectangles A–F are shown in Figure 9.

**Figure 9:**
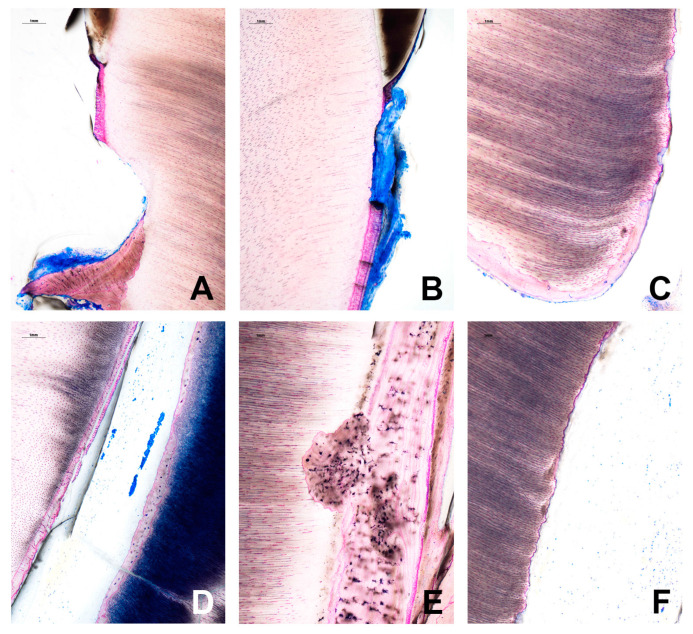
Higher power image of an explanted/extracted tooth 18 months after transplantation. (**A**,**B**) Resorptive lacunae and cementum damage on the root surface. (**C**,**D**) A layer of bone-like tissue is directly deposited on the canal dentin walls at the apical section of the root. (**E**) Dentin resorption. Osteocytes with slender, cytoplasmic processes radiating in all direction are present in the bone-like tissue. (**F**) Odontoblast-like cells are not found in the canal dentin walls.

A specialist in pathology assessed the stained section. Histology confirmed the presence of fibrovascular connective tissue in the root canal (Figure 8 and Figure 9). Resorptive lacunae and cementum damage were observed on the root surface (Figure 9A,B). At the apical section of the root, a layer of bone-like tissue was directly deposited on the canal dentin walls (Figure 9C,D) with focal dentin resorption (also known as replacement resorption, Figure 9D,E). In addition, osteocytes with slender cytoplasmic processes extending in all directions were present in the bone-like tissue (Figure 9E). In contrast, odontoblasts (Figure 9E) and cementoblast-like cells were absent.

## 3. Discussion

Revascularization occurred in a transplanted mature third molar after ATMT-EORER, proven by contrast-enhanced MRI 4 weeks after surgery. MRI brought immediate clarity on the PRV process in the tooth and could eliminate the risk of missing the early radiological signs of root resorption.

The application of the modern possibilities of cross-sectional imaging data is essential for a high success rate of the autotransplantation of teeth. CBCT- or MSCT-derived dicom data is used to create replicas of the transplant. The use of these replicas for recipient site preparation ensures two key factors for successful periodontal healing: minimal extraoral time and optimal fitting of the transplant, hence minimal damage of the active cells of the root surface. MRI on the other hand, has proven capable of showing revascularization and endodontic healing at an early stage, which is decisive for the further therapeutic procedure.

Display of pulpal perfusion with MRI has already been described in 2001 by Ploder et al. [7] using a 1.0 Tesla unit. Shortly after, Kress et al. [18] demonstrated that it was possible to produce MR images showing the perfusion of dental pulp in vivo, but that the analysis solely composed of non-contrast-enhanced sequences was not conclusive. They reported that the comparison of signal intensities before and after contrast agent administration showed the most significant difference between vital and avital teeth.

Assaf et al. [10] concluded that by application of 3T, PRV can be adequately visualized using fsT1w and fsT2w sequences, even without the use of contrast media, with scanning times of up to 20 min. They applied this protocol after replantation of avulsed teeth in children. There is a considerable risk that the pulp of the transplanted teeth may necrotize without adequate blood supply, therefore the MRI without contrast was able to detect avital teeth well. In our daily clinical setting, patients are examined in 30-min slots, limiting the maximum duration of the examination. For this reason, it was not feasible to perform native, high-resolution sequences lasting up to 20 min. Another consideration is that the longer the image acquisition time, the greater the chance that there will be motion artefacts.

Furthermore, MRI does not provide interpretable results if artefact causing elements are present. Especially orthodontic devices and dental implants cause major distortions. [19] Allergy to the contrast agent and claustrophobia are further contraindications. The application of contrast agents in children especially might raise concerns. In our facility, only macrocyclic gadolinium-based MRI contrast agents in single doses are used. Usually, patients are only examined once. In the MRI machines for pediatric examinations, Gadobutrol (Gadovist^®^; Bayer Healthcare, Leverkusen, Germany) is applied. So far, patients have experienced solely mild (mild itching) but no severe side effects.

All children are informed about the examination and possible side effects of MRI and MRI contrast media effects in the presence of their parents or guardians. In addition, known allergies and kidney disease are considered as contraindications. Several recent studies are consistent with our findings regarding the high safety of gadolinium-based macrocyclic MRI contrast agents [20,21,22].

In future studies, it would be interesting to evaluate the extent to which the vascular supply of the pulp can be determined using arterial spin labelling (ASL). This method would theoretically allow the measurement of local blood perfusion/supply without the need for intravenous contrast administration. A major limitation in imaging transplanted teeth is the small volume of the area examined at submillimeter resolution and, consequently, the length of the sequences applied. This is compounded by the small amount of signaling tissue in the oral area, especially in the pulp.

**Figure 10:**
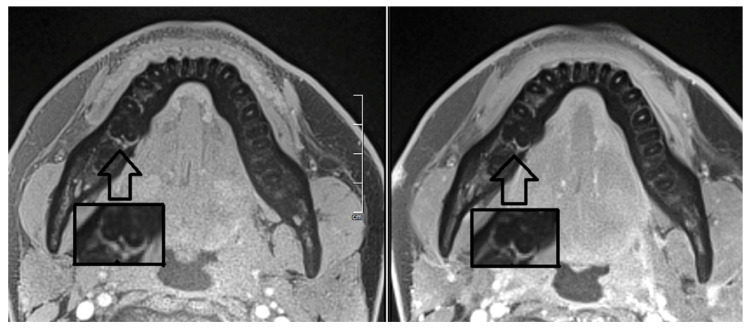
T1-weighted MRI on our 3T scanner, left without contrast and right with contrast. There is no significant T1 signal enhancement in the pulp in both native and post-contrast imaging. Note: Healthy teeth show clear and measurable contrast enhancement after magnification. The black arrow shows the region of interest with the image below magnified.

**Figure 11:**
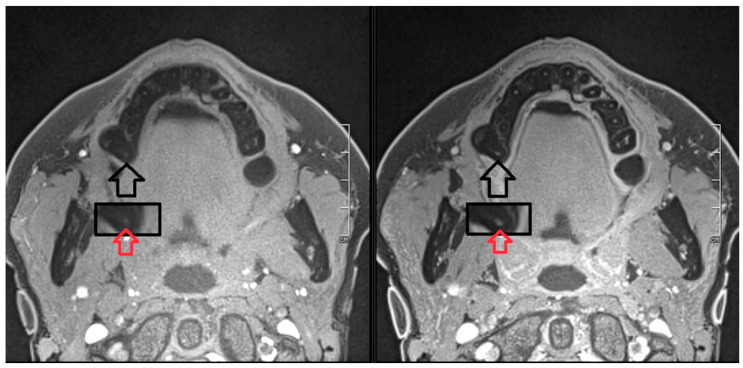
T1-weighted 3T MRI left, without, right, with contrast enhancement in the same patient at our hospital. The enhancement is clearly visible as a white spot in the pulp area after magnification on the right image. The black arrow shows the region of interest, the red arrow on the magnified image shows the pulp without and with contrast enhancement.

In our case, each motion reduced 3D T1 sequence took about 6:52 min, despite a 64-channel head coil and a 3T MRI machine. In patients with avital pulp, there was no measurable signal enhancement in the T1 sequence after gadolinium administration (Figure 10). In comparison, vital re-implanted teeth showed significant contrast uptake, similar to the other healthy teeth (Figure 11). It is to be expected that older and 1.5T machines with non-optimized coils will take significantly longer to produce reasonably good images with a good signal-to-noise ratio. Nevertheless, it is a good method for examining the vitality of the transplanted teeth and is well tolerated by our patients.

The transplanted tooth developed a resorptive lesion beneath the buccal cervical margin. Given the configuration of this lesion, it was obviously caused by trauma to the root cementum through the use of luxators during extraction and it worsened the prognosis for this case. In consultation with the patient, it was decided to remove the tooth. Consequently, the opportunity arose to have the transplant examined histologically.

ATMT-EORER has been described in animal studies [23,24] and was first implemented in humans by Jakse et al. [11]. To date, there have been only few reports regarding similar procedures and the mechanisms underlying pulpal healing have not been clarified yet. The histological assessment of an autotransplanted mature human tooth demonstrating these mechanisms is unique. Revascularization is mostly studied in animal trials, predominantly in studies addressing regenerative endodontics (RET). A review by Fang et al. [1] identified only two studies with histological examinations of intracanal tissues after RET [25,26]. Nevertheless, revascularization is a critical issue after transplantation, as its failure usually leads to endodontic infection and rapid external root resorption and possibly loss of the transplant.

Tooth germs with open apices have a greater ability to revascularize. This is clearly demonstrated by the consistently high success rates of pulpal healing in the autotransplantation of immature teeth with 2/3–3/4 developed roots [24,27,28,29].

Three mechanisms have to be considered to explain the highly predictable endodontic healing in immature transplants:

(a) A larger diameter of the apical foramen facilitates cell migration and ingrowth of new vessels into the ischemic pulp space.
(b) Immature teeth display a shorter root; therefore, the migration distance for the ingrown tissue is short.
(c) Immature teeth are endowed with more and a larger spectrum of stem cells.

The influence of stem/progenitor cells has been widely discussed in regenerative endodontic treatment (RET), where the term revascularization refers to the ingrowth of new tissue into a formerly infected root canal. Therefore, bleeding is provoked from the periapical tissue by introducing instruments below the apical constriction. The technique delivers undifferentiated mesenchymal stem cells into the root canal systems of adult patients with mature teeth [30].

In RET, these stem/progenitor cells might be derived from the periodontal ligament (PDL), apical papilla, Hertwig epithelial root sheath, or may be bone marrow mesenchymal stem cells or even surviving dental pulp stem cells [31].

After autotransplantation with root-end resection and hence the removal of the apical papilla, bone marrow stem cells, possibly triggered by the preparation of the recipient site, and stem cells from the PDL most likely drove the intracanal repair process in the present study. The use of tooth replicas for recipient site preparation significantly reduces the extraoral time of the transplant; as a consequence, it may be a key factor in the preservation of vital PDL cells for revascularization. Axin2 expressing cells have been identified in PDL [32]. Axin2+ mesenchymal PDL cells were described as key progenitor cell sources and might play a vital role in postnatal cementogenesis [33].

The mechanism of cell homing [3,34,35] addresses the availability of feasible stem cells beyond local availability. This aspect needs to be investigated in future studies.

The extracted molar subjected to histologic evaluation showed no cementum-like tissue in the revascularized root canals, but bone-like tissue with osteocytes in lacunae. This finding is consistent with the literature [36]. In our case, intracanal cementum was not observed. However, studies on the histologic features of revascularized teeth are rare, with animal trials predominantly animal trials addressing RET [2].

An early study by Skoglund and Tronstad [37] showed that the odontoblastic layer rarely survived in transplanted dog teeth, which was corroborated by Yamauchi et al. [38] after investigating RET in dog teeth. The mineralized tissue inside the canal was characterized by intracanal cement (IC) and bony islands. Wang et al. [39] identified IC, bony islands, and fibrous connective tissue (intracanal periodontal ligament) and hypothesized that different tissue types co-exist in the canal lumen. Furthermore, they reported the presence of lymphocyte infiltration next to IC and concluded that inflammation might stimulate the differentiation of stem/progenitor cells into cementoblasts.

The presented histology indicates that in cases of ATMT- EORER, pulp canal obliteration is presumably caused by the ingrowth of IC and not by reparative dentin. Still, the result is a functional tooth entailing no need for root canal treatment. Pulp canal obliteration is a sure sign of revascularization. Still, it takes several months until it can be proofed on radiographs. By then an endodontic infection may already have destroyed the transplant through rapid external infection-related root resorption. As contrast-enhanced-MRI can bring immediate clarity as soon as four weeks after surgery, it represents a preferable method that does not involve any additional harm to the patient. With an examination time of about 7 min for the T1 sequence, it can be easily integrated into clinical routine and evidence of reperfusion can reduce the number of check-ups required. Nevertheless, the application of MRI for dental purposes is confined by the limited availability of modern machines and highly skilled staff. For this reason, the routine application of the method can currently only be carried out at specialized facilities. Picturing the vascular supply of the pulp with other methods like arterial spin labelling (ASL) to avoid the need for intravenous contrast administration may be a matter of future research and developments.

A further shortcoming of the presented study is its limited perspective of the histological investigation, as only one case is assessed. In addition, relevant details may have been lost during cutting.

## 4. Conclusions

If applicable, contrast-enhanced MRI is a feasible method to demonstrate PRV of transplanted teeth and thus to ensure a good prognosis, even of mature transplanted teeth. Histologically, the intracanal tissue of the presented tooth showed no odontoblasts, but fibrovascular connective tissue. These results indicate that PRV in transplanted root-end resected teeth is driven by bone marrow stem cells and stem cells from the periodontal ligament. Likewise, pulp canal obliteration is presumably caused by the ingrowth of intracanal cementum or bone and not by reparative dentin.

## Data Availability

The data that support the findings of this study are available from the corresponding author upon reasonable request. The data are not publicly available due to privacy or ethical restrictions.

## References

[1] Verweij J.P., van Westerveld K.J.H., Anssari Moin D., Mensink G., van Merkesteyn J.P.R. (2020). Autotransplantation With a 3-Dimensionally Printed Replica of the Donor Tooth Minimizes Extra-Alveolar Time and Intraoperative Fitting Attempts: A Multicenter Prospective Study of 100 Transplanted Teeth. J. Oral. Maxillofac. Surg..

[2] Fang Y., Wang X., Zhu J., Su C., Yang Y., Meng L. (2018). Influence of apical diameter on the outcome of regenerative endodontic treatment in teeth with pulp necrosis: A review. J. Endod..

[3] Kim J.Y., Xin X., Moioli E.K., Chung J., Lee C.H., Chen M., Fu S.Y. (2010). Regeneration of dental-pulp-like tissue by chemotaxis—Induced cell homing. Tissue Eng. A.

[4] Wolf T.G., Kim P., Campus G., Stiebritz M., Siegrist M., Briseño-Marroquín B. (2020). 3-Dimensional analysis and systematic review of root canal morphology and physiological foramen geometry of 109 mandibular first premolars by micro-computed tomography in a mixed Swiss-German population. J. Endod..

[5] Kokai S., Kanno Z., Koike S., Uesugi S., Takahashi Y., Ono T., Soma K. (2015). Retrospective study of 100 autotransplanted teeth with complete root formation and subsequent orthodontic treatment. Am. J. Orthod. Dentofac. Orthop..

[6] Tsukiboshi M. (2002). Autotransplantation of teeth: Requirements for predictable success. Dent. Traumatol..

[7] Ploder O., Partik B., Rand T., Fock N., Voracek M., Undt G., Baumann A. (2001). Reperfusion of autotransplanted teeth—Comparison of clinical measurements by means of dental magnetic resonance imaging. Oral. Surg. Oral. Med. Oral. Pathol. Oral. Radiol. Endodontology.

[8] Nakashima M., Akamine A. (2005). The Application of Tissue Engineering to Regeneration of Pulp and Dentin in Endodontics. J. Endod..

[9] Annibali S., Bellavia D., Ottolenghi L., Cicconetti A., Cristalli M.P., Quaranta R., Pilloni A. (2013). Micro-CT and PET analysis of bone regeneration induced by biodegradable scaffolds as carriers for dental pulp stem cells in a rat model of calvarial “critical size” defect: Preliminary data. J. Biomed. Mater. Res. Part. B Appl. Biomater..

[10] Assaf A.T., Zrnc T.A., Remus C.C., Khokale A., Habermann C.R., Schulze D., Fiehler J., Heiland M., Sedlacik J., Friedrich R.E. (2015). Early detection of pulp necrosis and dental vitality after traumatic dental injuries in children and adolescents by 3-Tesla magnetic resonance imaging. J. Cranio Maxillofac. Surg..

[11] Jakse N., Ruckenstuhl M., Rugani P., Kirnbauer B., Sokolowski A., Ebeleseder K. (2018). Influence of extraoral apicoectomy on revascularization of an autotransplanted tooth: A case report. J. Endod..

[12] Gaviño Orduña J.F., García García M., Dominguez P., Caviedes Bucheli J., Martin Biedma B., Abella Sans F., Manzanares Céspedes M.C. (2020). Successful pulp revascularization of an autotransplantated mature premolar with fragile fracture apicoectomy and plasma rich in growth factors: A 3-year follow-up. Int. Endod. J..

[13] Raabe C., Bornstein M.M., Ducommun J., Sendi P., Von Arx T., Janner S.F.M. (2021). A retrospective analysis of autotransplanted teeth including an evaluation of a novel surgical technique. Clin. Oral. Investig..

[14] Rugani P., Kirnbauer B., Mischak I., Ebeleseder K., Jakse N. (2022). Extraoral Root-End Resection May Promote Pulpal Revascularization in Autotransplanted Mature Teeth—A Retrospective Study. J. Clin. Med..

[15] Nagendrababu V., Chong B.S., McCabe P., Shah P.K., Priya E., Jayaraman J., Pulikkotil S.J., Dummer P.M.H. (2020). PRICE 2020 guidelines for reporting case reports in Endodontics: Explanation and elaboration. Int. Endod. J..

[16] Donath K. (1998). [The separating thin section technique for the production of histological preparations from tissues and materials that cannot be sectioned: Description of apparatus and methods] German. Die Trenn-Dünnschliff-Technik zur Herstellung histologischer Präparate von nicht schneidbaren Geweben und Materialien. Präparator.

[17] Laczko J., Levai G. (1975). A simple differential staining method for semi-thin sections of ossyfying cartilage and bone tissue embedded in epoxy resin. Mikroskopie.

[18] Kress B., Buhl Y., Anders L., Stippich C., Palm F., Bähren W., Sartor K. (2004). Quantitative analysis of MRI signal intensity as a tool for evaluating tooth pulp vitality. Dentomaxillofac. Radiol..

[19] Reda R., Zanza A., Mazzoni A., Cicconetti A., Testarelli L., Di Nardo D. (2021). An Update of the Possible Applications of Magnetic Resonance Imaging (MRI) in Dentistry: A Literature Review. J. Imaging.

[20] Shah R., D’Arco F., Soares B., Cooper J., Brierley J. (2019). Use of gadolinium contrast agents in paediatric population: Donald Rumsfeld meets Hippocrates!. Br. J. Radiol..

[21] Prince M.R., Lee H.G., Lee C.H., Youn S.W., Lee I.H., Yoon W., Yang W., Wang H., Wang J., Shih T.T. (2017). Safety of gadobutrol in over 23,000 patients: The GARDIAN study, a global multicentre, prospective, non-interventional study. Eur. Radiol..

[22] Heshmatzadeh Behzadi A., McDonald J. (2022). Gadolinium-based contrast agents for imaging of the central nervous system: A multicenter European prospective study. Medicine.

[23] Skoglund A. (1981). Pulpal changes in replanted and autotransplanted apicoectomized mature teeth of dogs. Int. J. Oral. Surg..

[24] Skoglund A. (1981). Vascular changes in replanted and autotransplanted apicoectomized mature teeth of dogs. Int. J. Oral. Surg..

[25] Nosrat A., Kolahdouzan A., Hosseini F., Mehrizi E.A., Verma P., Torabinejad M. (2015). Histologic outcomes of uninfected human immature teeth treated with regenerative endodontics: 2 case reports. J. Endod..

[26] Martin G., Ricucci D., Gibbs J.L., Lin L.M. (2013). Histological findings of revascularized/revitalized immature permanent molar with apical periodontitis using platelet-rich plasma. J. Endod..

[27] Andreasen J.O., Paulsen H.U., Yu Z., Schwartz O. (1990). A long-term study of 370 autotransplanted premolars. Part III. Periodontal healing subsequent to transplantation. Eur. J. Orthod..

[28] Filippi A. (2008). [Tooth transplantation] German. Quintessenz.

[29] Paulsen H.U., Andreasen J.O., Schwartz O. (1995). Pulp and periodontal healing, root development and root resorption subsequent to transplantation and orthodontic rotation: A long-term study of autotransplanted premolars. Am. J. Orthod. Dentofac. Orthop..

[30] Chrepa V., Henry M.A., Daniel B.J., Diogenes A. (2015). Delivery of Apical Mesenchymal Stem Cells into Root Canals of Mature Teeth. J. Dent. Res..

[31] Lei L., Chen Y., Zhou R., Huang X., Cai Z. (2015). Histologic and Immunohistochemical Findings of a Human Immature Permanent Tooth with Apical Periodontitis after Regenerative Endodontic Treatment. J. Endod..

[32] Yuan X., Pei X., Zhao Y., Tulu U.S., Liu B., Helms J.A. (2018). A Wnt-Responsive PDL Population Effectuates Extraction Socket Healing. J. Dent. Res..

[33] Xie X., Wang J., Wang K., Li C., Zhang S., Jing D., Xu C., Wang X., Zhao H., Feng J.Q. (2019). Axin2+-Mesenchymal PDL Cells, Instead of K14+ Epithelial Cells, Play a Key Role in Rapid Cementum Growth. J. Dent. Res..

[34] Eramo S., Natali A., Pinna R., Milia E. (2018). Dental pulp regeneration via cell homing. Int. Endod. J..

[35] Ahmed G.M., Abouauf E.A., AbuBakr N., Fouad A.M., Dörfer C.E., Fawzy El-Sayed K.M. (2021). Cell-Based Transplantation versus Cell Homing Approaches for Pulp-Dentin Complex Regeneration. Stem Cells Int..

[36] Yanpiset K., Trope M. (2000). Pulp revascularization of replanted immature dog teeth after different treatment methods. Endod. Dent. Traumatol..

[37] Skoglund A., Tronstad L. (1981). Pulpal changes in replanted and autotransplanted immature teeth of dogs. J. Endod..

[38] Yamauchi N., Nagaoka H., Yamauchi S., Teixeira F.B., Miguez P., Yamauchi M. (2011). Immunohistological characterization of newly formed tissues after regenerative procedure in immature dog teeth. J. Endod..

[39] Wang X., Thibodeau B., Trope M., Lin L.M., Huang G.T.-J. (2010). Histologic characterization of regenerated tissues in canal space after the revitalization/revascularization procedure of immature dog teeth with apical periodontitis. J. Endod..

